# Supplementary material for: Content-rich biological network constructed by mining PubMed abstracts
Source: BMC Bioinformatics. 2004 Oct 8;5:147. doi: 10.1186/1471-2105-5-147 (PMC528731; doi:10.1186/1471-2105-5-147)
Supplement: Additional File 2 — The original results of the above study (non-essential files are deleted to keep the file size under the limit set by BMC bioinformatics). [file 1471-2105-5-147-S2.bz2 › chilibotAdditionalFile2/dip20/612ID7791872E10995/html/RAF1_RAP1A.html]

 


 **RAF1** and **RAP1A** 
  
Found 29 abstracts in PubMed, retrieved 20.  
 

 What does Google say? 
 PDF only 
| .edu only 

---

**Interactive relationship** (e.g. stimulation, inhibition, etc)

**Inhibitory relationship**- These results suggest that  **Rap1A**  interferes with Ras dependent Raf 1  [ **RAF1** ]  activation by inhibiting binding of Ras to Raf 1  [ **RAF1** ]  CRR.  Ref: 9115221 J Biol Chem, 1997
- These results indicate that the CRR binding activity and the Ras suppressive function of  **Rap1A**  can be modulated through phosphorylation and suggest that  **Rap1A**  may function as a PKA dependent regulator of Raf 1  [ **RAF1** ]  activation, not merely as a suppressor.  Ref: 9867809 J Biol Chem, 1999
- The interaction between  **Rap1A**  and RGF RBD shows similar characteristics to the Ras Raf  [ **RAF1** ]  interaction because it is blocked by mutations in the effector region D38A and it inhibits the dissociation of guanine nucleotide, which is the basis for the quantitative measurements in this work.  Ref: 8636102 J Biol Chem, 1996
- The effect of  **Rap1A**  V12 was dramatically potentiated by the concurrent activation of PKC, the cAMP pathway, and Ras, and this potentiation was blocked by dominant negative mutants of Ras and Raf  [ **RAF1** ] .  Ref: 12473665 J Biol Chem, 2003
**Neutral relationship**- ... H Ras binds with high affinity KD = 20 nM to Raf  [ **RAF1** ]  RBD and with low affinity KD = 1 microM to RGF RBD,  **Rap1A**  shows the opposite behavior.  Ref: 8636102 J Biol Chem, 1996
- Differential interaction of the ras family GTP binding proteins H Ras,  **Rap1A** , and R Ras with the putative effector molecules Raf  [ **RAF1** ]  kinase and Ral guanine nucleotide exchange factor.  Ref: 8636102 J Biol Chem, 1996
- Finally, a B Raf  [ **RAF1** ]  CRD mutant whose interaction with  **Rap1A**  is selectively enhanced was isolated and found to possess the double mutation K252E M278T.  Ref: 10454553 Mol Cell Biol, 1999
- The Ras and  **Rap1A**  proteins can bind to the Raf  [ **RAF1** ]  and RalGDS families.  Ref: 9516482 J Biol Chem, 1998
- Using the selective interactions of Ras and Krev 1  [ **RAP1A** ]   **Rap1A**  with Raf  [ **RAF1** ] , RalGDS, and Krit1 as a model, we systematically compared LexA and cI fused baits and reporters.  Ref: 10358061 J Biol Chem, 1999
- ... B Raf  [ **RAF1** ] , whose CRD is replaced by Raf 1  [ **RAF1** ]  CRD, lost its response to  **Rap1A** .  Ref: 10454553 Mol Cell Biol, 1999
- The Ras homologue  **Rap1A**  is incapable of activating Raf 1  [ **RAF1** ]  and even antagonizes Ras dependent activation of Raf 1  [ **RAF1** ] .  Ref: 10454553 Mol Cell Biol, 1999
- The importance of the interaction at the CRD is further supported by a domain shuffling experiment between Raf 1  [ **RAF1** ]  and B Raf  [ **RAF1** ] , which clearly indicated that the nature of CRD determines the specificity of response to  **Rap1A**  Raf 1  [ **RAF1** ] , whose CRD is replaced by B Raf  [ **RAF1** ]  CRD, became activatable by  **Rap1A** .  Ref: 10454553 Mol Cell Biol, 1999
- Both  **Rap1A**  and Ha Ras E31K mutant failed to activate Raf 1  [ **RAF1** ]  and interfered with Ha Ras dependent activation of Raf 1  [ **RAF1** ]  in Sf9 cells.  Ref: 9115221 J Biol Chem, 1997
- Of the two cDNA classes isolated, one contained a single isolate encoding the known Krev 1  [ **RAP1A** ]  interactor Raf  [ **RAF1** ] , while the second contained multiple isolates coding for a previously undescribed protein which we have designated Kritl for Krev Interaction Trapped 1.  Ref: 9285558 Oncogene, 1997
- On the other hand, B Raf  [ **RAF1** ] , another Raf  [ **RAF1** ]  family member, is activatable by both Ras and  **Rap1A** .  Ref: 10454553 Mol Cell Biol, 1999
- Coassociation of  **Rap1A**  and Ha Ras with Raf 1  [ **RAF1** ]  N terminal region interferes with ras dependent activation of Raf 1  [ **RAF1** ] .  Ref: 9115221 J Biol Chem, 1997
- All of these results clearly show that the sharp contrast between the characteristics of Ras and  **Rap1A** , with respect to the interactions with Raf 1  [ **RAF1** ]  and RalGDS, depends on their residues at position 31.  Ref: 9516482 J Biol Chem, 1998
- These results indicate that the strength of interaction with Ras and  **Rap1A**  at its CRD may be a critical determinant of regulation of the Raf  [ **RAF1** ]  kinase activity by the Ras family small GTPases.  Ref: 10454553 Mol Cell Biol, 1999
- The complex of Ras with the Ras binding domain RBD of Raf  [ **RAF1** ]  has been modelled by homology to the E30D, K31E  **Rap1A**  RBD complex, and both have been subjected to multiple molecular dynamics simulations in solution.  Ref: 10090289 Proteins, 1999
- In the complex of the Ras like E30D K31E mutant  **Rap1A**  with the Ras binding domain RBD, residues 51 131 of Raf 1  [ **RAF1** ] , Glu31 in  **Rap1A**  forms a tight salt bridge with Lys84 in Raf 1  [ **RAF1** ] .  Ref: 9931261 J Mol Biol, 1999
- Here, the  **Rap1A**  like D30E E31K mutant of Ras was prepared and shown to bind the Raf 1  [ **RAF1** ]  RBD less strongly than wild type Ras, but slightly more tightly than the E31K mutant.  Ref: 9931261 J Mol Biol, 1999
- However,  **Rap1A** , which has an identical effector domain to that of Ras, cannot activate Raf 1  [ **RAF1** ]  and even antagonizes several Ras functions in vivo.  Ref: 9115221 J Biol Chem, 1997
- Enhanced binding of  **Rap1A**  to CRR led to co association of  **Rap1A**  and Ha Ras with Raf 1  [ **RAF1** ]  N terminal region through binding to CRR and Ras binding domain, respectively.  Ref: 9115221 J Biol Chem, 1997
- We proposed previously that this property of  **Rap1A**  may be attributable to its greatly enhanced interaction with Raf 1  [ **RAF1** ]  CRD compared to Ras.  Ref: 10454553 Mol Cell Biol, 1999
- We have previously shown that this suppressive activity of  **Rap1A**  is attributable to its greatly enhanced ability to bind to the cysteine rich region CRR, residues 152 184 of Raf 1  [ **RAF1** ]  compared with that of Ras.  Ref: 9867809 J Biol Chem, 1999

**Non-interactive relationship** (e.g. studied together, co-existance, homology, etc.)

- This is done by taking the complex between  **Rap1A**  Rap and the p21ras binding domain of c Raf  [ **RAF1** ]  Raf  [ **RAF1** ]  RBD Nassar et al.  Ref: 9533625 Proteins, 1998
- Effect of phosphorylation on activities of  **Rap1A**  to interact with Raf 1  [ **RAF1** ]  and to suppress Ras dependent Raf 1  [ **RAF1** ]  activation.  Ref: 9867809 J Biol Chem, 1999
- The interactions of H Ras, R Ras, and  **Rap1A**  with the Ras binding domains RBD of the c Raf  [ **RAF1** ]  kinase and of the Ral guanine nucleotide exchange factor RGF was studied biochemically in solution.  Ref: 8636102 J Biol Chem, 1996
- In cells expressing the activated  **Rap1A**  protein, a PI3K Raf  [ **RAF1** ]  MEK dependent pathway mediates transient ERK activation.  Ref: 11146560 Oncogene, 2000
- B Raf  [ **RAF1** ]  carrying this mutation was not activated by  **Rap1A**  but retained its response to Ras.  Ref: 10454553 Mol Cell Biol, 1999
- Surprisingly, as opposed to  **Rap1A** , which has no effect on Raf 1  [ **RAF1** ]  oncogene mediated transformation, C3G also reduced dramatically 6 8 fold the number of v raf  [ **RAF1** ]  induced foci in transfected NIH3T3 cells.  Ref: 9482107 Oncogene, 1998
- Correspondingly, RasG12V E31K as well as  **Rap1A** , but not RasG12V E31R, exhibited abnormally tight binding with the cysteine rich domain of Raf 1  [ **RAF1** ] .  Ref: 9516482 J Biol Chem, 1998
- When interactions with Ras and  **Rap1A**  were measured, B Raf  [ **RAF1** ]  CRD did not exhibit the enhanced interaction with  **Rap1A** , suggesting that the strength of interaction at CRDs may account for the differential action of  **Rap1A**  on Raf 1  [ **RAF1** ]  and B Raf  [ **RAF1** ] .  Ref: 10454553 Mol Cell Biol, 1999
- However, it is not known whether  **Rap1A**  phosphorylation is involved in regulation of its cellular functions, including suppression of Ras dependent Raf 1  [ **RAF1** ]  activation.  Ref: 9867809 J Biol Chem, 1999
- Furthermore, a mutant  **Rap1A**  S180E, whose sole PKA phosphorylation residue, Ser 180, was substituted by an acidic residue, Glu, to mimic its phosphorylated form, failed to suppress Ras dependent Raf 1  [ **RAF1** ]  activation in COS 7 cells.  Ref: 9867809 J Biol Chem, 1999
